# Neoadjuvant Therapy for Resectable Pancreatic Cancer: An Evolving Paradigm Shift

**DOI:** 10.3389/fonc.2019.01085

**Published:** 2019-10-17

**Authors:** Akhil Chawla, Cristina R. Ferrone

**Affiliations:** Harvard Medical School, Massachusetts General Hospital, Boston, MA, United States

**Keywords:** pancreatic adenocarcinoma, resectable, neoadjuvant, adjuvant, chemotherapy

## Abstract

Non-metastatic pancreatic adenocarcinoma (PDAC) is associated with a high rate of recurrence and lethality. In addition, less than half of all patients are able to complete systemic therapy after curative-intent pancreatectomy. With its well-known potential benefits, this report highlights the current prospective data relevant to the use of neoadjuvant systemic therapy in resectable PDAC. Recently, there have been numerous reports, many of which consist of long-awaited multi-intuitional trial data evaluating the use of neoadjuvant systemic chemotherapy in non-metastatic PDAC as well as the use of combination chemotherapy regimens in the adjuvant setting. Currently, recommended guidelines for neoadjuvant systemic therapy only exist for borderline-resectable and locally-advanced disease. Given the plethora of new data, there has been a shift in the paradigm of how resectable pancreatic cancer is treated at certain centers across the world. This review highlights the relevant available data from recent sentinel prospective trials and how they relate to the systemic treatment of resectable PDAC in the neoadjuvant setting.

Pancreatic adenocarcinoma (PDAC) is currently the 3rd most common cause of cancer death in the United States (1). Surgical resection offers the only chance of cure. Long-term survival rates after the diagnosis of PDAC is dismal, with less than a tenth of patients alive after 5 years. A strategy involving neoadjuvant therapy aims to improve the chance of a long-term cure (1). The well-recognized radiologic classification scheme used for PDAC has been broadly adopted and is currently used as a model to guide therapy by the NCCN (2). The NCCN defines Resectable disease as tumor with no arterial contact (celiac axis, superior mesenteric artery, or common hepatic artery) and no venous tumor contact with the superior mesenteric vein or portal vein. If there is venous contact, there must be ≤180° of contact without any vein contour irregularity to qualify as Resectable (2).

Landmark clinical trials have demonstrated a survival benefit with the addition of chemotherapy to curative-intent pancreatectomy. The Gastrointestinal Study Group was the first to demonstrate a survival benefit for adjuvant chemoradiation in pancreatic cancer (3). The ESPAC-1 trial showed benefit for the addition of chemotherapy for patients treated with resection using 6 months of fluorouracil. These patients had a 5-year survival rate of 21 vs. 8% in those who did not receive adjuvant therapy (4). The CONKO-001 trial demonstrated that 6 months of gemcitabine treatment in the adjuvant setting increased both overall survival (OS) and disease-free survival (DFS) for patients with resected PDAC (5). The ESPAC-4 trial also demonstrated evidence for the benefit of adjuvant chemotherapy, suggesting further benefit with combining gemcitabine and capecitabine (6).

These initial trials, all in the adjuvant setting, demonstrated modest improvements in DFS and OS (5–8). However, nearly half of patients fail to complete chemotherapy after pancreatectomy (3). This has led to an increased emphasis on the use of neoadjuvant chemotherapy for patients without evidence of metastatic disease. Neoadjuvant chemotherapy has multiple potential benefits including early treatment of occult micrometastatic disease, improved compliance with chemotherapy, the potential to downstage tumors and increase margin-negative resection rates, as well as select patients who are likely to benefit from surgery (4, 5). Given these advantages, there is enthusiasm to evaluate the use neoadjuvant therapy in the upfront resectable population.

Although resectable PDAC by radiologic criteria demonstrates no clinical evidence of distant disease, up to 17% have occult metastatic disease identified at the time of the operation (9) and over 70% of patients are found to have nodal metastases on pathology after resection (10). In addition, <7% achieve long-term cure after curative-intent pancreatectomy (11, 12). These data favor the principal that the majority of resectable PDAC patients have occult micrometastatic disease at presentation. The current standard of care for patients with resectable PDAC involves an operation followed by adjuvant chemotherapy (13). The use of neoadjuvant therapy for resectable pancreatic cancer has been a topic of debate with multiple reports evaluating the utility of this strategy (9, 14). A propensity matched analysis of over 15,000 patients with resectable PDAC from the National Cancer Database demonstrated that neoadjuvant therapy has a significant survival benefit in early-stage, resected pancreatic head adenocarcinoma (15). However, as the generalizability of retrospective reports are clearly limited by selection bias, prospective data is needed to evaluate this strategy in the upfront resectable disease population. In line with this, preliminary results of trials evaluating neoadjuvant therapy in a mixed population of resectable and borderline-resectable PDAC have recently been reported.

Preliminary results from the PREOPANC-1 randomized phase III trial have shown that patients with resectable and borderline resectable PDAC perform better in terms of survival if gemcitabine-based therapy is given in the neoadjuvant vs. adjuvant setting (6). In total, 246 patients were randomized to adjuvant gemcitabine (arm A) vs. perioperative gemcitabine (arm B). Patients in arm A received six cycles of gemcitabine after surgery. In arm B, patients received two cycles of gemcitabine, plus gemcitabine-based chemoradiation at a dose of 36 Gy/2.4 per fraction over 3 weeks followed by an operation followed by four more cycles of adjuvant gemcitabine. The primary endpoint of this trial is OS. While complete data and analyses from this trial have not yet been reported, preliminary results from this trial demonstrated an OS benefit for arm B at a median of 29.9 vs. 16.8 months for arm A (*p* < 0.001) (10). This trial demonstrates early level-one evidence for the benefit of a perioperative strategy in delivering chemotherapy with single-agent gemcitabine.

The JSAP-05 randomized multi-institutional phase II/III trial evaluated neoadjuvant chemotherapy with gemcitabine and S-1, an oral fluoropyrimidine derivative (11), vs. upfront surgery in patients deemed to have resectable or borderline-resectable PDAC (12). Patients enrolled in this trial were diagnosed with histologically-confirmed resectable or borderline-resectable PDAC, which was defined by lack of arterial involvement, and randomized to the perioperative arm which consisted of neoadjuvant-chemotherapy with two cycles of gemcitabine and S-1 followed by surgery, followed by four more cycles of adjuvant S-1, or to the adjuvant arm which treated patients with upfront surgery and four cycles of adjuvant S-1. In the phase-II portion of this trial, the primary endpoint was R0-resection rate and was found to be equivalent in both groups (13). The primary endpoint for the phase-III portion of the trial is OS. In total, 364 patients were enrolled from 57 institutions. The median OS for the perioperative group was 36.7 vs. 26.6 months in the adjuvant group reaching a hazard ratio of 0.72 (95% CI 0.55–0.94; *p* = 0.015) favoring perioperative therapy with equivalent operative morbidity between the two groups. As we await a formalized report, these findings point to the premise that the use of neoadjuvant therapy may be beneficial for patients with resectable PDAC.

In addition to the appropriate setting during which to deliver chemotherapy, the optimal chemotherapeutic regimen has yet to be determined. Multi-agent chemotherapy regimens such as 5-FU, oxaliplatin, and irinotecan (FOLFIRINOX) and nab-paclitaxel plus gemcitabine have been prospectively shown to enhance DFS and OS over single-agent regimens in the metastatic setting (15, 16). The recently reported PRODIGE24/CCTG PA.6 trial demonstrated that modified FOLFIRINOX (mFOLFIRINOX) is superior to gemcitabine in the adjuvant setting (2). The median OS in patients randomized after resection to receive 24 weeks of adjuvant mFOLFIRINOX (12 cycles) was 54.4 months vs. a median OS of 35.0 months in patients randomized to receive 24 weeks of single-agent gemcitabine (6 cycles). The median DFS, which was the primary endpoint of this trial, was 21.6 vs. 12.8 months in favor of the mFOLFIRINOX arm. Although those enrolled in this trial were a select group of patients who had already undergone an R0 or R1 curative-intent resection and eligible only if the serum CA 19-9 level was <180 U per milliliter, these unprecedented survival outcomes support the use of FOLFIRINOX as a standard adjuvant therapy regimen for patients who can tolerate the associated toxicity (17, 18).

The combination of gemcitabine and nab-paclitaxel has increasingly gained popularity as well. Initial data supporting its superiority over single-agent gemcitabine in the metastatic setting prompted a clinical trial evaluating this question for the adjuvant setting. The unanticipated results of the phase III APACT trial demonstrated no significant difference in the primary endpoint of DFS evaluated by independent reviewer when comparing patients who were randomized to gemcitabine plus nab-paclitaxel vs. gemcitabine alone (19). Given these striking results, we await the results SWOG-1505 trial which has completed accrual. This trial is evaluating perioperative mFOLFIRINOX vs. gemcitabine/nab-paclitaxel (12 weeks pre-operatively and 12 weeks post-operatively in both arms) in patients with resectable PDAC (20).

Neoadjuvant FOLFIRINOX has been shown to decrease nodal positivity, increase margin-negative resection rates, and increases OS in a retrospective analysis of borderline resectable and locally advanced patients (21). A recent phase II trial from the Massachusetts General Hospital demonstrated that eight cycles of neoadjuvant FOLFIRINOX plus radiotherapy in borderline resectable patients was well-tolerated in over 80% and led to margin-negative resection rates in >90% in those who underwent resection (14). The two-year DFS and OS survival for these borderline-resectable patients was 43% and 56%, respectively. Another phase-II trial from Massachusetts General Hospital evaluated neoadjuvant FOLFRINOX with losartan, a potent inhibitor of the TGF-beta pathway (22), plus radiotherapy, in a population of locally-advanced patients. In this trial, 31 of 34 patients achieved the primary endpoint of R0-resection (23). After a median follow-up of 17.1 months, the median DFS and OS were 17.5 and 31.4 months, respectively. These outcomes are exceptional for a population of patients with locally-advanced PDAC. Taken together, both of these single-arm trials highlight the conceivable benefits of neoadjuvant FOLFIRINOX in terms of survival and facilitating margin-negative resections in borderline and locally-advanced PDAC.

In order to infer the value of neoadjuvant FOLFORINOX to the resectable pancreatic cancer space, a definitive phase-III randomized controlled trial clinical trial is necessary comparing a neoadjuvant vs. adjuvant strategy. This question is of great equipoise and is an area that urgently needs prospective investigation. Even with modern surgical techniques and contemporary systemic therapy, margin-negative pancreatectomy followed by standard adjuvant chemotherapy leads to unacceptably high rates of recurrence with a small minority of patients achieving long-term survival. The potential down-side to treating all patients with resectable PDAC with neoadjuvant chemotherapy is the plausible over-treatment of patients with systemic therapy and unnecessarily subjecting patients to its associated toxicities. In addition, there is a theoretical potential for missing a “window” for curative intent-resection in patients with clearly resectable disease. However, proponents of neoadjuvant therapy suggest that patients who ultimately progress during neoadjuvant therapy are those that would not have derived benefit from pancreatectomy. Finally, patients with obstructive jaundice who are to be treated with a neoadjuvant approach are likely to require biliary stenting. This adds an additional layer of complexity as pre-operative stenting has been shown to increase the incidence of overall complications and wound infection after pancreatectomy (24). However, recent data suggests that when treatment of obstructive jaundice cannot be avoided, a longer period of time elapsed from the time of biliary stent placement to pancreatectomy may reduce morbidity and post-operative complications (25).

Many unanswered questions remain. For example, given the additive toxicity seen with combination chemotherapy, we must identify the appropriate length of time or number of cycles to deliver treatment, whether it be in the neoadjuvant, perioperative, or adjuvant setting. Furthermore, the utility of radiation in PDAC is not entirely clear. How this relates to the benefits of systemic therapy is not currently known and remains to be elucidated. The role of radiation therapy in PDAC is a focus of current ongoing trials (26–28). As we evolve through a new era of multimodality treatment for PDAC, there is great emphasis on further understanding the molecular and genetic alterations in pancreatic cancer and how this relates to treatment response. Advances in this field will undoubtedly lead to more sophisticated techniques of assessing treatment response as well as individualized methods to guide treatment.

## Author Contributions

AC was involved in writing of this article. CF was involved in the writing and editing of this article.

### Conflict of Interest

The authors declare that the research was conducted in the absence of any commercial or financial relationships that could be construed as a potential conflict of interest.
